# 8,10-Diiodo-2,6-dioxo-4λ^3^-ioda-3,5-dioxatricyclo­[5.3.1.0^4,11^]undeca-1(11),7,9-triene-9-carb­oxy­lic acid

**DOI:** 10.1107/S1600536812005351

**Published:** 2012-02-17

**Authors:** Daopeng Sheng, Lu Han, Yi Zhang, Yanzhao Yang

**Affiliations:** a Key Laboratory for Special Functional Aggregated Materials of the Education Ministry, School of Chemistry and Chemical Engineering, Shandong University, Jinan 250100, People’s Republic of China

## Abstract

In the title compound, C_9_HI_3_O_6_·2H_2_O, the mol­ecule is located on a twofold axis that gives rise to disorder of the carboxyl group. This disorder is correlated with the disorder of one of the H atoms of the water mol­ecule. The carboxyl group is twisted relative to the attached benzene ring by 75.1 (4)°. The intra­molecular I⋯O distance is 2.112 (6) Å. Mol­ecules are linked *via* O—H⋯O hydrogen bonding, C—I⋯O halogen bonding, with I⋯O distances in the range 3.156 (5)–3.274 (6) Å, and dipolar C=O⋯C=O inter­actions between the carboxyl and carboxyl­ate groups, with an O⋯C distance of 2.944 (10) Å.

## Related literature

For general background to 1,3,5-triiodo­benzene derivatives, see: Morin *et al.* (1987 ▶); Yu & Watson (1999 ▶). For information on the related compound 1,3,5-triiodo-2,4,6-trimethyl­benzene, see: Bosch & Barnes (2002 ▶); Boudjada *et al.* (2001 ▶); Reddy *et al.* (2006 ▶). For the crystal structures of 5-amino-2,4,6-triiodo­isophthalic acid monohydrate and 5-amino-2,4,6-triiodo­isophthalic acid–4,4′-bipyridine *N*,*N*′-dioxide–water (1/1/1), see: Beck & Sheldrick (2008 ▶); Zhang *et al.* (2011 ▶).
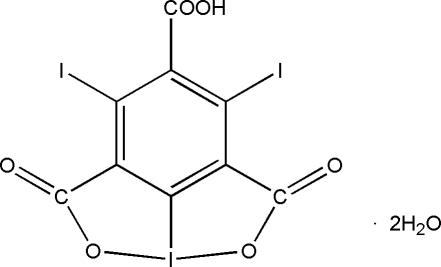


## Experimental

### 

#### Crystal data

C_9_HI_3_O_6_·2H_2_O
*M* *_r_* = 621.83
Monoclinic, 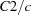

*a* = 14.7667 (8) Å
*b* = 11.9890 (6) Å
*c* = 9.7419 (5) Å
β = 127.4236 (5)°
*V* = 1369.68 (12) Å^3^
*Z* = 4
Mo *K*α radiation
μ = 6.88 mm^−1^
*T* = 130 K
0.32 × 0.14 × 0.12 mm

#### Data collection

Bruker APEXII CCD area-detector diffractometer
Absorption correction: multi-scan (*APEX2*; Bruker, 2007 ▶) *T*_min_ = 0.217, *T*_max_ = 0.492
4078 measured reflections
1547 independent reflections
1515 reflections with *I* > 2σ(*I*)
*R*_int_ = 0.016

#### Refinement

*R*[*F* ^2^ > 2σ(*F* ^2^)] = 0.042
*wR*(*F* ^2^) = 0.097
*S* = 1.23
1547 reflections
93 parameters
H-atom parameters constrained
Δρ_max_ = 2.36 e Å^−3^
Δρ_min_ = −2.23 e Å^−3^

### 

Data collection: *APEX2* (Bruker, 2007 ▶); cell refinement: *SAINT* (Bruker, 2007 ▶); data reduction: *SAINT*; program(s) used to solve structure: *SIR97* (Altomare *et al.*, 1999 ▶); program(s) used to refine structure: *SHELXL97* (Sheldrick, 2008 ▶); molecular graphics: *DIAMOND* (Brandenburg, 1999 ▶); software used to prepare material for publication: *publCIF* (Westrip, 2010 ▶).

## Supplementary Material

Crystal structure: contains datablock(s) I, global. DOI: 10.1107/S1600536812005351/gk2447sup1.cif

Structure factors: contains datablock(s) I. DOI: 10.1107/S1600536812005351/gk2447Isup2.hkl

Supplementary material file. DOI: 10.1107/S1600536812005351/gk2447Isup3.cml

Additional supplementary materials:  crystallographic information; 3D view; checkCIF report

## Figures and Tables

**Table 1:** Hydrogen-bond geometry (Å, °)

*D*—H⋯*A*	*D*—H	H⋯*A*	*D*⋯*A*	*D*—H⋯*A*
O3—H3⋯O1*W*	0.82	2.08	2.772 (9)	142
O1*W*—H1*W*⋯O3	0.82	1.98	2.772 (9)	163
O1*W*—H2*W*⋯O1*W*^i^	0.82	1.94	2.730 (14)	160
O1*W*—H3*W*⋯O1^ii^	0.82	2.24	3.053 (9)	172

Symmetry codes: (i) 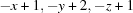; (ii) 
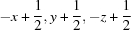
.

**Fig. 1.**
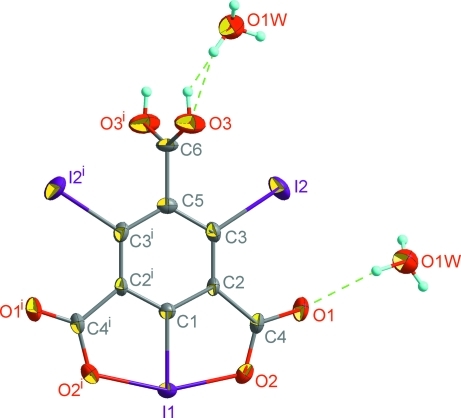
Molecular structure of the title compound. Displacement ellipsoids are drawn at the 50% probability level. Symmetry code: (i) 1 - x, y, 1.5 + z.

**Fig. 2.**
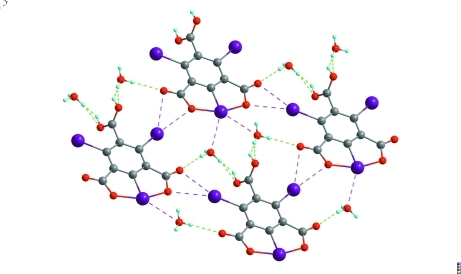
Partial view of the crystal structure. Molecules are linked by O—H···O (green dashed lines) and O—H···I hydrogen bonds (purple dashed lines).

**Fig. 3.**
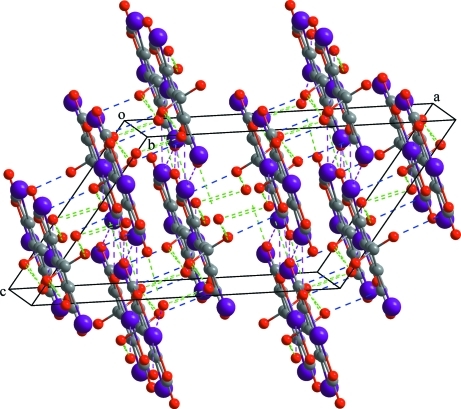
Packing of the title compound, hydrogen atoms are omitted for clarity. Hydrogen bonds (green), halogen bonds (purple) and dipolar interaction (blue) are shown as dashed lines.

## supplementary crystallographic information

### Comment

Iodine-based compounds have always been used as contrast agents for X-ray imaging (Morin *et al.*, 1987). The 1,3,5-triiodo-benzene core has been the basis of many contrast agents (Yu & Watson, 1999). In this paper, we present the crystal structure of a new compound based on 1,3,5-triiodobenzene core.

In the title compound the organic molecule is located on a twofold axis what results in disorder of the carboxylic group (Fig. 1). In the crystal structure, there are hydrogen bonds between symmetry related water molecules as well as between the water molecule and the carboxylic group. It indicates that one of the hydrogen atoms of the water molecule has to be disordered and this disorder is evidently correlated with the disorder of the carboxylic group. The hydrogen atom and the oxygen atom forming hydrogen bond between the water molecule and the carboxylic group are either from the water molecule or the carboxylic group. There is also a hydrogen bond between the water molecule and the carboxylate O1 atom. Hydrogen atom involved in this interaction has full occupancy. The dihedral angle between the plane of the carboxyl group and the benzene ring is 75.1 (4)°.

In addition to hydrogen bond, the structure is stabilized by halogen bonding between the I2 atom and the carboxylate group O1 and O2 atoms. There is also a halogen bond between the water molecule and I1 atom (Fig. 2). A dipolar interaction between carboxyl C6—O3 and carboxylate C4 also is observed (C···O 2.95 Å) (Fig. 3).

### Experimental

A mixture of 1,3,5-triiodo-2,4,6-trimethylbenzene (5 g) and excess of potassium permanganate (80 g) was dissolved in pyridine (60 ml) and heated under reflux for 24 h to produce the title compound (m.p. 573 K, decompose). Crystallization was carried out from a mixture of water and methanol (*v*/*v* 1:2). Colorless crystals suitable for X-ray single-crystal diffraction were obtained by slow evaporation method.

### Refinement

H atom of the carboxylic group was placed in geometrically calculated position and refined using a riding model the the occupantion factor of 0.5. Positions of H atoms from the water molecule were calculated after analysis of possible hydrogen-bond interactions. The occupantion factors of H1W and H2W were assigned as 0.5.

The isotropic displacement parameters of all H atoms were set to 1.5 times the equivalent displacement parameter of their parent O atoms.

### Crystal data

**Table:**

C_9_HI_3_O_6_·2H_2_O	*F*(000) = 1128
*M**_r_* = 621.83	*D*_x_ = 3.016 Mg m^−^^3^
Monoclinic, *C*2/*c*	Melting point: 573 K
Hall symbol: -C 2yc	Mo *K*α radiation, λ = 0.71069 Å
*a* = 14.7667 (8) Å	Cell parameters from 3350 reflections
*b* = 11.9890 (6) Å	θ = 2.4–27.4°
*c* = 9.7419 (5) Å	µ = 6.88 mm^−^^1^
β = 127.4236 (5)°	*T* = 130 K
*V* = 1369.68 (12) Å^3^	Prism, colourless
*Z* = 4	0.32 × 0.14 × 0.12 mm

### Data collection

**Table:**

Bruker APEXII CCD area-detector diffractometer	1547 independent reflections
Radiation source: fine-focus sealed tube	1515 reflections with *I* > 2σ(*I*)
Graphite monochromator	*R*_int_ = 0.016
φ and ω scans	θ_max_ = 27.4°, θ_min_ = 2.4°
Absorption correction: multi-scan (*APEX2*; Bruker, 2007)	*h* = −10→19
*T*_min_ = 0.217, *T*_max_ = 0.492	*k* = −14→15
4078 measured reflections	*l* = −12→12

### Refinement

**Table:**

Refinement on *F*^2^	Primary atom site location: structure-invariant direct methods
Least-squares matrix: full	Secondary atom site location: difference Fourier map
*R*[*F*^2^ > 2σ(*F*^2^)] = 0.042	Hydrogen site location: inferred from neighbouring sites
*wR*(*F*^2^) = 0.097	H-atom parameters constrained
*S* = 1.23	*w* = 1/[σ^2^(*F*_o_^2^) + (0.0157*P*)^2^ + 40.7765*P*] where *P* = (*F*_o_^2^ + 2*F*_c_^2^)/3
1547 reflections	(Δ/σ)_max_ < 0.001
93 parameters	Δρ_max_ = 2.36 e Å^−^^3^
0 restraints	Δρ_min_ = −2.23 e Å^−^^3^

### Special details

**Table:**

Geometry. All esds (except the esd in the dihedral angle between two l.s. planes) are estimated using the full covariance matrix. The cell esds are taken into account individually in the estimation of esds in distances, angles and torsion angles; correlations between esds in cell parameters are only used when they are defined by crystal symmetry. An approximate (isotropic) treatment of cell esds is used for estimating esds involving l.s. planes.
Refinement. Refinement of *F*^2^ against ALL reflections. The weighted *R*-factor *wR* and goodness of fit *S* are based on *F*^2^, conventional *R*-factors *R* are based on *F*, with *F* set to zero for negative *F*^2^. The threshold expression of *F*^2^ > σ(*F*^2^) is used only for calculating *R*-factors(gt) *etc*. and is not relevant to the choice of reflections for refinement. *R*-factors based on *F*^2^ are statistically about twice as large as those based on *F*, and *R*- factors based on ALL data will be even larger.

### Fractional atomic coordinates and isotropic or equivalent isotropic displacement parameters (Å^2^)

**Table:**

	*x*	*y*	*z*	*U*_iso_*/*U*_eq_	Occ. (<1)
C1	0.5000	0.3381 (8)	0.7500	0.0216 (19)	
C2	0.4147 (5)	0.3903 (6)	0.5979 (8)	0.0207 (13)	
C3	0.4155 (6)	0.5066 (6)	0.5979 (9)	0.0249 (14)	
C4	0.3340 (6)	0.3120 (6)	0.4528 (9)	0.0248 (14)	
C5	0.5000	0.5629 (8)	0.7500	0.0222 (19)	
C6	0.5000	0.6916 (8)	0.7500	0.027 (2)	
I1	0.5000	0.16946 (5)	0.7500	0.02526 (18)	
I2	0.29502 (5)	0.59994 (5)	0.37929 (8)	0.0469 (2)	
O1	0.2532 (5)	0.3419 (5)	0.3093 (7)	0.0386 (14)	
O2	0.3559 (5)	0.2049 (5)	0.4946 (7)	0.0313 (12)	
O3	0.5338 (6)	0.7375 (5)	0.6751 (9)	0.0474 (16)	
H3	0.5308	0.8054	0.6818	0.071*	0.50
O1W	0.4370 (6)	0.9449 (6)	0.5383 (9)	0.0494 (16)	
H1W	0.4763	0.8910	0.5958	0.074*	0.50
H2W	0.4756	0.9896	0.5303	0.074*	0.50
H3W	0.3831	0.9240	0.4416	0.074*	

### Atomic displacement parameters (Å^2^)

**Table:**

	*U*^11^	*U*^22^	*U*^33^	*U*^12^	*U*^13^	*U*^23^
C1	0.021 (4)	0.019 (4)	0.021 (5)	0.000	0.011 (4)	0.000
C2	0.017 (3)	0.024 (3)	0.012 (3)	0.000 (2)	0.004 (2)	−0.001 (2)
C3	0.021 (3)	0.030 (4)	0.017 (3)	0.005 (3)	0.008 (3)	0.004 (3)
C4	0.021 (3)	0.031 (4)	0.015 (3)	−0.002 (3)	0.007 (3)	−0.003 (3)
C5	0.026 (5)	0.018 (4)	0.029 (5)	0.000	0.020 (4)	0.000
C6	0.039 (6)	0.012 (4)	0.035 (6)	0.000	0.025 (5)	0.000
I1	0.0282 (3)	0.0169 (3)	0.0269 (3)	0.000	0.0148 (3)	0.000
I2	0.0427 (4)	0.0392 (3)	0.0341 (3)	0.0125 (2)	0.0104 (3)	0.0160 (2)
O1	0.031 (3)	0.042 (3)	0.019 (3)	0.000 (3)	0.003 (2)	−0.004 (2)
O2	0.030 (3)	0.029 (3)	0.024 (3)	−0.008 (2)	0.011 (2)	−0.008 (2)
O3	0.074 (5)	0.028 (3)	0.069 (5)	0.000 (3)	0.058 (4)	0.005 (3)
O1W	0.050 (4)	0.044 (4)	0.048 (4)	−0.008 (3)	0.026 (3)	−0.002 (3)

### Geometric parameters (Å, º)

**Table:**

C1—C2^i^	1.380 (8)	C5—C3^i^	1.400 (8)
C1—I1	2.022 (9)	C5—C6	1.543 (13)
C2—C3	1.394 (10)	C6—O3	1.237 (7)
C2—C4	1.501 (9)	I1—O2	2.113 (5)
C3—C5	1.400 (8)	O3—H3	0.8200
C3—I2	2.085 (7)	O1W—H1W	0.8201
C4—O1	1.216 (9)	O1W—H2W	0.8200
C4—O2	1.326 (9)	O1W—H3W	0.8201
			
C4···O3^ii^	2.944 (10)	I2···O2^iv^	3.156 (5)
I1···O1W^iii^	3.173 (7)	I2···O1^iv^	3.274 (6)
			
C2^i^—C1—C2	126.1 (9)	C3—C5—C3^i^	122.3 (9)
C2^i^—C1—I1	117.0 (5)	C3—C5—C6	118.8 (5)
C2—C1—I1	117.0 (5)	O3^i^—C6—O3	127.1 (10)
C1—C2—C3	116.8 (6)	O3—C6—C5	116.5 (5)
C1—C2—C4	114.3 (6)	C1—I1—O2	78.38 (15)
C3—C2—C4	128.9 (6)	O2^i^—I1—O2	156.8 (3)
C2—C3—C5	119.0 (7)	C4—O2—I1	116.1 (4)
C2—C3—I2	122.3 (5)	C6—O3—H3	109.5
C5—C3—I2	118.7 (6)	H1W—O1W—H2W	109.6
O1—C4—O2	121.6 (7)	H1W—O1W—H3W	109.5
O1—C4—C2	124.1 (7)	H2W—O1W—H3W	109.6
O2—C4—C2	114.2 (6)		
			
C2^i^—C1—C2—C3	0.5 (5)	I2—C3—C5—C3^i^	−179.6 (5)
I1—C1—C2—C3	−179.5 (5)	C2—C3—C5—C6	−179.5 (5)
C2^i^—C1—C2—C4	179.7 (6)	I2—C3—C5—C6	0.4 (5)
I1—C1—C2—C4	−0.3 (6)	C3—C5—C6—O3^i^	105.1 (5)
C1—C2—C3—C5	−0.9 (9)	C3—C5—C6—O3	−74.9 (5)
C4—C2—C3—C5	180.0 (6)	C3^i^—C5—C6—O3	105.1 (5)
C1—C2—C3—I2	179.1 (4)	C2^i^—C1—I1—O2	179.6 (4)
C4—C2—C3—I2	0.1 (11)	C2—C1—I1—O2	−0.4 (4)
C1—C2—C4—O1	179.7 (7)	O1—C4—O2—I1	180.0 (6)
C3—C2—C4—O1	−1.3 (13)	C2—C4—O2—I1	−1.5 (8)
C1—C2—C4—O2	1.2 (9)	C1—I1—O2—C4	1.1 (5)
C3—C2—C4—O2	−179.7 (7)	O2^i^—I1—O2—C4	1.1 (5)
C2—C3—C5—C3^i^	0.5 (5)		

Symmetry codes: (i) −*x*+1, *y*, −*z*+3/2; (ii) −*x*+1, −*y*+1, −*z*+1; (iii) *x*, *y*−1, *z*; (iv) −*x*+1/2, *y*+1/2, −*z*+1/2.

### Hydrogen-bond geometry (Å, º)

**Table:**

*D*—H···*A*	*D*—H	H···*A*	*D*···*A*	*D*—H···*A*
O3—H3···O1*W*	0.82	2.08	2.772 (9)	142
O1*W*—H1*W*···O3	0.82	1.98	2.772 (9)	163
O1*W*—H2*W*···O1*W*^v^	0.82	1.94	2.730 (14)	160
O1*W*—H3*W*···O1^iv^	0.82	2.24	3.053 (9)	172

Symmetry codes: (iv) −*x*+1/2, *y*+1/2, −*z*+1/2; (v) −*x*+1, −*y*+2, −*z*+1.
